# Supplementary material for: A Critical View of the Application of the APEX Software (Aqueous Photochemistry of Environmentally-Occurring Xenobiotics) to Predict Photoreaction Kinetics in Surface Freshwaters
Source: Molecules. 2019 Dec 18;25(1):9. doi: 10.3390/molecules25010009 (PMC7017383; doi:10.3390/molecules25010009)
Supplement: Supplementary file 1 [file molecules-25-00009-s001.zip › 3.2.4_gcc-4.4.0.zip!/3.2.4_gcc-4.4.0/3.2.4_gcc-4.4.0/doc/octave/html/interpreter/Function-Files.html]

Function Files - Untitled


Next: Script Files,
Previous: Default Arguments,
Up: Functions and Scripts


---

### 11.7 Function Files

Except for simple one-shot programs, it is not practical to have to
define all the functions you need each time you need them. Instead, you
will normally want to save them in a file so that you can easily edit
them, and save them for use at a later time.

Octave does not require you to load function definitions from files
before using them. You simply need to put the function definitions in a
place where Octave can find them.

When Octave encounters an identifier that is undefined, it first looks
for variables or functions that are already compiled and currently
listed in its symbol table. If it fails to find a definition there, it
searches a list of directories (the path) for files ending in
.m that have the same base name as the undefined
identifier.^1^ Once Octave finds a file with a name that matches,
the contents of the file are read. If it defines a *single*
function, it is compiled and executed. See Script Files, for more
information about how you can define more than one function in a single
file.

When Octave defines a function from a function file, it saves the full
name of the file it read and the time stamp on the file. If the time
stamp on the file changes, Octave may reload the file. When Octave is
running interactively, time stamp checking normally happens at most once
each time Octave prints the prompt. Searching for new function
definitions also occurs if the current working directory changes.

Checking the time stamp allows you to edit the definition of a function
while Octave is running, and automatically use the new function
definition without having to restart your Octave session.

To avoid degrading performance unnecessarily by checking the time stamps
on functions that are not likely to change, Octave assumes that function
files in the directory tree
octave-home/share/octave/version/m
will not change, so it doesn't have to check their time stamps every time the
functions defined in those files are used. This is normally a very good
assumption and provides a significant improvement in performance for the
function files that are distributed with Octave.

If you know that your own function files will not change while you are
running Octave, you can improve performance by calling
`ignore_function_time_stamp ("all")`, so that Octave will
ignore the time stamps for all function files. Passing
`"system"` to this function resets the default behavior.


— Command: edit name  
— Command: edit field value  
— Command: value = **edit** get field  
> Edit the named function, or change editor settings.
>
> If `edit` is called with the name of a file or function as
> its argument it will be opened in a text editor.
>
> - If the function name is available in a file on your path and
>   that file is modifiable, then it will be edited in place. If it
>   is a system function, then it will first be copied to the directory
>   `HOME` (see further down) and then edited.
>   If no file is found, then the m-file
>   variant, ending with ".m", will be considered. If still no file
>   is found, then variants with a leading "@" and then with both a
>   leading "@" and trailing ".m" will be considered.- If name is the name of a function defined in the interpreter but
>     not in an m-file, then an m-file will be created in `HOME`
>     to contain that function along with its current definition.- If `name.cc` is specified, then it will search for `name.cc`
>       in the path and try to modify it, otherwise it will create a new
>       .cc file in `HOME`. If name happens to be an
>       m-file or interpreter defined function, then the text of that
>       function will be inserted into the .cc file as a comment.- If name.ext is on your path then it will be edited, otherwise
>         the editor will be started with HOME/name.ext as the
>         filename. If name.ext is not modifiable, it will be copied to
>         `HOME` before editing.
>
>         **WARNING!** You may need to clear name before the new definition
>         is available. If you are editing a .cc file, you will need
>         to mkoctfile name.cc before the definition will be available.
>
> If `edit` is called with field and value variables,
> the value of the control field field will be value.
> If an output argument is requested and the first argument is `get`
> then `edit` will return the value of the control field field.
> If the control field does not exist, edit will return a structure
> containing all fields and values. Thus, `edit get all` returns
> a complete control structure.
> The following control fields are used:
>
> editor: This is the editor to use to modify the functions. By default it uses Octave's `EDITOR` built-in function, which comes from `getenv("EDITOR")` and defaults to `emacs`. Use `%s` In place of the function name. For example, [EDITOR, " %s"]: Use the editor which Octave uses for `bug_report`. "xedit %s &": pop up simple X11 editor in a separate window "gnudoit -q \"(find-file \\\"%s\\\")\"": Send it to current Emacs; must have `(gnuserv-start)` in .emacs. See also field 'mode', which controls how the editor is run by Octave. On Cygwin, you will need to convert the Cygwin path to a Windows path if you are using a native Windows editor. For example ``` '"C:/Program Files/Good Editor/Editor.exe" "$(cygpath -wa %s)"' ``` home: This is the location of user local m-files. Be be sure it is in your path. The default is ~/octave. author: This is the name to put after the "## Author:" field of new functions. By default it guesses from the `gecos` field of password database. email: This is the e-mail address to list after the name in the author field. By default it guesses `<$LOGNAME@$HOSTNAME>`, and if `$HOSTNAME` is not defined it uses `uname -n`. You probably want to override this. Be sure to use `<user@host>` as your format. license: gpl: GNU General Public License (default). bsd: BSD-style license without advertising clause. pd: Public domain. "text": Your own default copyright and license. Unless you specify pd, edit will prepend the copyright statement with "Copyright (C) yyyy Function Author". mode: This value determines whether the editor should be started in async mode (editor is started in the background and Octave continues) or sync mode (Octave waits until the editor exits). Set it to "async" to start the editor in async mode. The default is "sync" (see also "system"). editinplace: Determines whether files should be edited in place, without regard to whether they are modifiable or not. The default is `false`.

— Built-in Function: **mfilename** ()  
— Built-in Function: **mfilename** (`"fullpath"`)  
— Built-in Function: **mfilename** (`"fullpathext"`)  
> Return the name of the currently executing file. At the top-level,
> return the empty string. Given the argument `"fullpath"`,
> include the directory part of the file name, but not the extension.
> Given the argument `"fullpathext"`, include the directory part
> of the file name and the extension.

— Built-in Function: val = **ignore\_function\_time\_stamp** ()  
— Built-in Function: old\_val = **ignore\_function\_time\_stamp** (new\_val)  
> Query or set the internal variable that controls whether Octave checks
> the time stamp on files each time it looks up functions defined in
> function files. If the internal variable is set to `"system"`,
> Octave will not automatically recompile function files in subdirectories of
> octave-home/lib/version if they have changed since
> they were last compiled, but will recompile other function files in the
> search path if they change. If set to `"all"`, Octave will not
> recompile any function files unless their definitions are removed with
> `clear`. If set to "none", Octave will always check time stamps
> on files to determine whether functions defined in function files
> need to recompiled.

- Manipulating the load path- Subfunctions- Private Functions- Overloading and Autoloading- Function Locking- Function Precedence

---

#### Footnotes

[1] The .m suffix was chosen for compatibility
with matlab.

---
